# Participatory science in action: Transforming urban soil research

**DOI:** 10.1016/j.isci.2025.112362

**Published:** 2025-04-23

**Authors:** Anna Paltseva

**Affiliations:** 1 Departments of Agronomy and Horticulture and Landscape Architecture at Purdue University, West Lafayette, IN 47907, USA

## Abstract

Anna Paltseva, PhD, is an urban soil scientist committed to improving soil health in city environments through research and community collaboration. Her work in New York City and Lafayette, Louisiana, demonstrates the power of participatory science to address urban soil contamination and promote environmental justice. Now, at Purdue University, Anna aims to expand these efforts, using tools like her Urban Soil Guide and X-ray fluorescence (XRF) analyzer to empower communities, increase awareness, and build resilient urban soil practices. This Backstory emphasizes the need for accessible, community-driven approaches to manage soil contamination in diverse urban settings.

**Figure:**
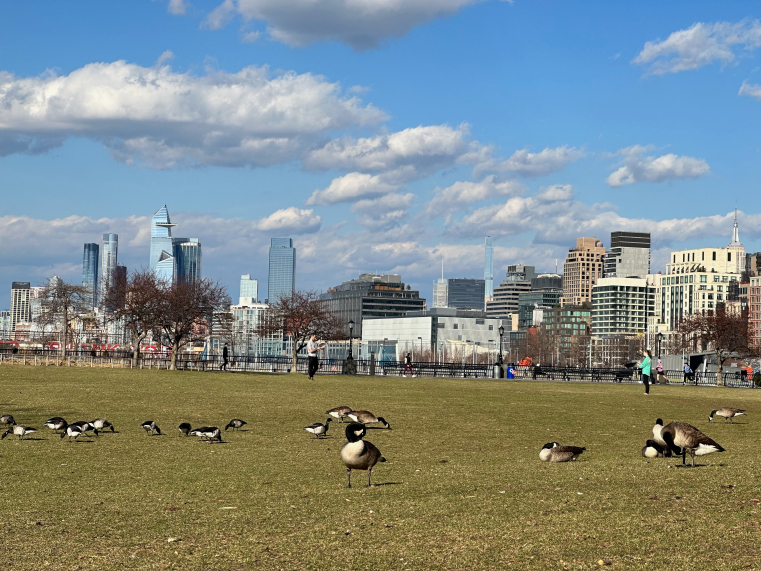
Above image: Urban landscapes of New York City (photo credit: Anna Paltseva).

This experience made Anna realize that urban soil science is most impactful when shared with the people who need the information the most.

A combination of community involvement, transparent communication, and institutional support helps build trust and foster a collaborative spirit within the community.

By fostering community involvement and leveraging technology, we can build more resilient urban environments together.

### Beginnings

#### Why urban soils matter

Soil science is the study of soil as a natural resource, exploring its formation, properties, and interactions with the environment. When we talk about urban soils, these are not just any soils—they are unique due to human influence and city infrastructure. Their characteristics are shaped by compaction, contamination, changes in water drainage, and altered organic matter.^1^ These features are important because urban soils often have higher concentrations of heavy metals, resulting from their proximity to buildings, roads, and industrial activity.

These distinct characteristics impact not just the soil itself but also the environment and the communities around it. Urban soils play crucial roles in filtering water, sequestering carbon, supporting biodiversity, reducing flood risks, stabilizing the foundations for buildings, and even helping regulate urban temperatures by mitigating the heat island effect.^2^ They also promote plant growth, which enhances green spaces and contributes to overall well-being. Yet, because these soils are so closely intertwined with human activity, challenges like contamination, erosion, and compaction can reduce their ability to provide vital ecosystem services.

This makes understanding and managing urban soils especially important. Participatory science—where everyday people participate in scientific research—can help by engaging communities to collect soil data, monitor environmental changes, and raise public awareness about the importance of urban soil health. This collaboration between scientists and the general public improves data collection and empowers communities to take an active role in caring for their urban environments.

### Motivation

#### Transforming soil research with community involvement

When Anna first moved to New York City in 2011, she was amazed by how many gardens she saw tucked throughout the city—full of tomatoes, squash, zucchini, cucumbers, and eggplants. At the time, she did not quite understand why people were growing food in a place with so many grocery stores and other resources. Only later did she come to appreciate the meaning and deeper purpose behind community gardens. A community garden is a volunteer-led garden for communal use that produces nutritious food for locals, encourages family bonding, reduces grocery bill, supports mental health benefits, minimizes criminal activities, and fosters a deeper connection to agriculture and nature.^3^

When Anna began her PhD studies at CUNY Graduate Center, with her home campus at CUNY Brooklyn College Urban Soils Lab, she discovered a significant interest of NYC residents in growing food and understanding soil in the city to improve their crops. Anna’s original research goal was to assess bioaccessible levels––fractions of metals that can be uptaken by an organism––of lead and arsenic in soils based on samples NYC residents sent to their lab for testing. She aimed to understand how these values correlated with total metal concentrations. Yet, their lab, led by Dr. Zhongqi Cheng, frequently received requests from the community for soil testing and remediation recommendations. As a result, her research expanded to include field experiments in both community gardens and suburban farms. This required close collaboration with local gardeners, as she needed access to their properties and their help with watering and monitoring the experimental plots—designated garden beds where soil treatments and measurements were conducted as part of the research.

A notable example is the Sterling Community Garden in Brooklyn, which faced significant attention regarding soil contamination. In 2014, the New York Post published articles highlighting concerns over “toxic soils” and “toxic plants” in this garden and others across the city.^4^^,^^5^ The articles included phrases like “disturbing new state data” and “this is insane,” which caused considerable worry among urban gardeners in New York City. In response to alarming media reports about contamination in the Sterling Community Garden, Brooklyn College Urban Soils Lab conducted soil testing and established experimental plots to assess actual risks—growing vegetables with three different soil amendments alongside control plots in the garden’s untreated soil. Spoiler alert: severe contamination was detected only in one area beneath peach trees, which was naturally unused for gardening due to excessive shade.^6^

**Figure:**
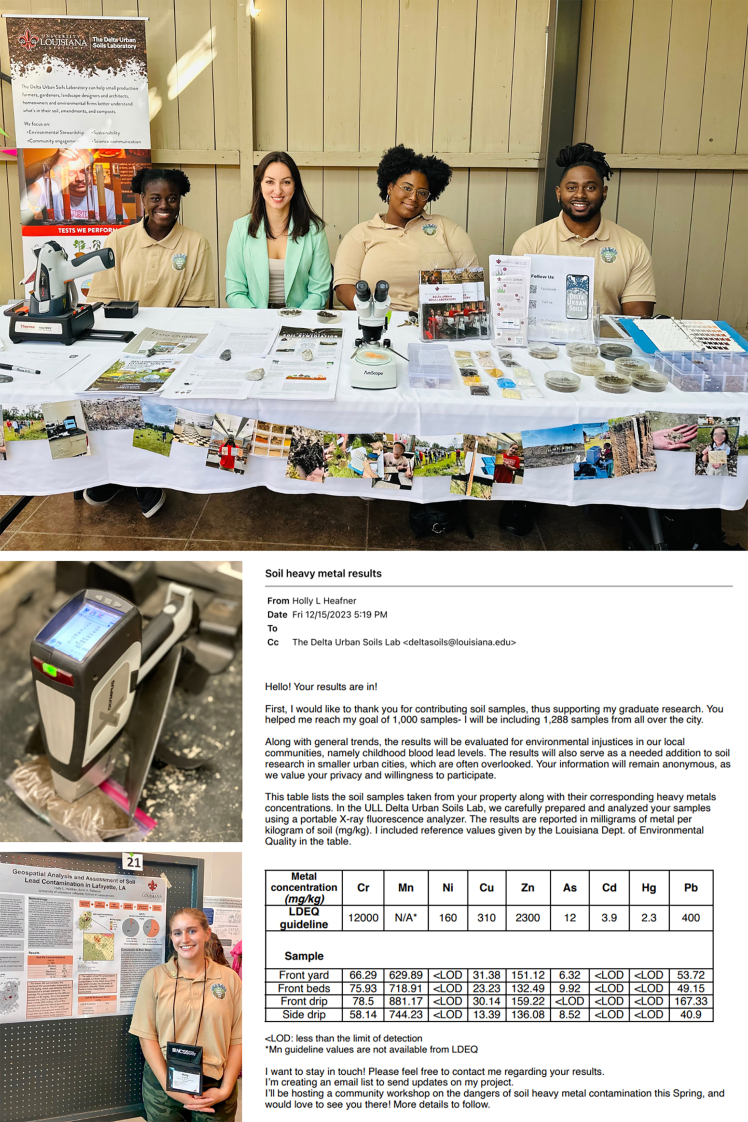
At community events, the Delta Urban Soils Lab team—Victoire Soumano, Anna Paltseva, Eriell Jenkins, and Tyrell Lassair (top image)—offers free soil testing at the New Orleans Plant Festival in 2023. The middle image shows an X-ray fluorescence (XRF) analyzer measuring a soil sample used in the studies. At the bottom, former graduate student Holly Heafner, who led the Lafayette research project and presented its findings at a soil conference in North Dakota, is pictured alongside an example of the soil report sent to residents who participated in the Lafayette, LA study (courtesy of Anna Paltseva and Holly Heafner).

In 2014, Anna started to lead workshops for agencies, garden festivals, and non-profit organizations, sharing her knowledge on soil contamination and remediation with the residents. For NYC Urban Soils Institute (USI), Anna developed educational materials on urban soils and interpretations. Anna also conducted train-the-trainer workshops and outdoor soil testing in parks and community gardens using a portable X-ray fluorescence (XRF) analyzer, which allowed for on-the-spot testing of soil samples for heavy metal contaminants. USI was invited to participate in community events by partner organizations, where participants brought soil samples from their properties and the researchers provided immediate XRF soil testing results with feedback. Alternatively, they would advertise soil testing services on social media and via email list. The initial reaction from the community gardeners was enthusiastic and curious. Many of them already possessed a significant amount of knowledge about gardening and were eager to learn more about soil and how its quality affects their crops and children’s health. This hands-on approach not only helped to collect valuable soil samples but also educated the public about soil contamination and its implications. When people received their results, especially those with high metal concentrations, they explained what the results meant and provided instructions for low-cost remediation methods, such as building raised beds or covering soil with compost or mulch.

Through this experience, Anna learned about the strong interest people have in soil health and their concerns about heavy metal concentrations, whether for growing produce in their backyards or preventing potential exposure to their children. Communicating and translating scientific findings to a layperson teaches the importance of in-person interactions and the crucial step of getting back to them with the results. This experience made Anna realize that urban soil science is most impactful when shared with the people who need the information the most. This principle applies across many scientific fields—research becomes truly impactful when it is communicated clearly, shared directly with communities, and used to address real-world concerns.

With these concepts in mind, in 2021, Anna founded the Delta Urban Soil Lab at the University of Louisiana at Lafayette, which focused on testing soils for communities in Louisiana and beyond. Because researchers cannot access private properties without permission, it is essential for residents to understand the importance of soil testing and remediation to volunteer for Anna’s research. They can also provide valuable information about their property’s land use history and management practices. The inclusion of residential soils in mapping significantly improves the quality of city mapping. Anna’s group shared the research findings with local authorities via email, published papers, personal communication, or presentations. For privacy reasons, the actual addresses of the findings are never shared. Here is an example of a dataset they collected in Lafayette, LA, with research funded by the Louisiana Board of Regents.

### Interdisciplinary science and collaborations

#### Bringing soil science to the people

For Anna, engaging people in her research involved a comprehensive approach to educating communities and providing essential resources. Her overarching research goal is to assess urban soil contamination and offer remediation strategies. In Louisiana, Anna’s lab focused heavily on outreach programs that featured workshops and hands-on testing events tailored to community needs. To engage local communities, Anna’s lab partnered with Lafayette Consolidated Government, community organizations, and small businesses—many led by University of Louisiana at Lafayette alumni—who helped connect the team with neighborhoods and supported outreach at community meetings. To educate the public about soil contamination risks, the Delta Urban Soils Lab team organized workshops, webinars, and interactive exhibits at local events such as Moncus Park’s Spring Fest, downtown Art Walks, and the Halloween Art and Nature Festival. At the same time, incorporating activities like soil painting with soil-based art supplies for children also helped attract parents and fostered a family-friendly environment. These events, held in accessible public spaces such as parks, nature reserves, and education centers, ensured broad community participation. To ensure that the educational materials and activities were accessible and understandable for all participants, Anna and her students wrote them using accessible language and with simplified diagrams and images, encouraged people to ask questions, and provided take-home materials. Lafayette residents were invited to submit their samples for free soil heavy metal testing funded by the Louisiana Board of Regents Grant.

Targeting low-income neighborhoods, particularly African American communities,^7^ was a key research focus for Anna’s team due to the prevalent environmental justice issues in these areas. Their research revealed that median soil lead levels were 50.5 mg/kg in predominantly Black neighborhoods compared to 23.5 mg/kg in predominantly White neighborhoods in Lafayette, LA, highlighting significantly higher lead contamination in Black-majority areas.^8^ These communities often contain older housing and infrastructure—conditions that tend to correlate with economic disadvantage, regardless of race—and are more likely to have elevated soil lead levels due to legacy contamination from past industrial activities, lead-based paint, and leaded gasoline. This points to an environmental justice issue, indicating that minority and economically challenged communities may face greater exposure to harmful soil contaminants.

**Figure:**
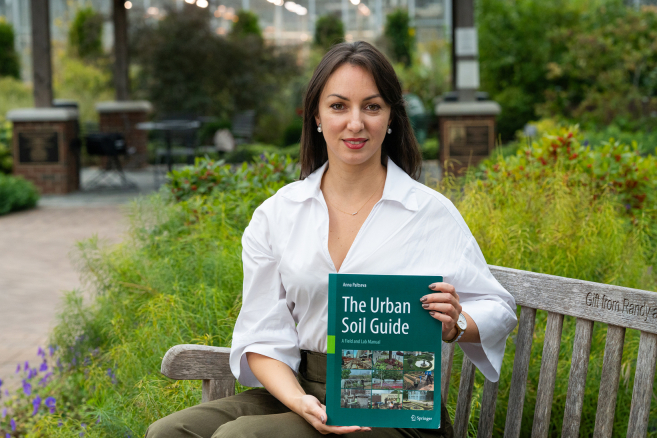
Anna Paltseva holds a copy of her Urban Soil Guide: A Field and Laboratory Manual on Purdue University campus (photo credit: Joshua Clark).

A combination of targeted outreach, transparent communication, and institutional support helped foster trust and a collaborative spirit across the community. The team also collaborated with local news outlets to raise awareness about the importance of soil testing and to encourage community involvement. Promoting the study at public events enabled the team to connect with residents across Lafayette and nearby areas.

#### Building trust within the community

In Lafayette, LA, the success of the 2-year soil testing project was driven by the enthusiastic participation of about 100 city residents, who contributed a total of 763 soil samples from their homes and public areas. These included a mix of samples brought in by residents and others collected directly from properties by graduate student Holly Heafner, who led the sampling efforts, soil testing, and data analyses. This level of participation is considered strong for urban soil studies, where sampling is often more labor-intensive than in other participatory science projects, which typically engage anywhere from a few dozen to a few hundred participants depending on context. To ensure sampling quality and mapping accuracy, they followed strict protocols, collecting multiple samples per site and recording GPS coordinates. They used Geographic Information System (GIS) tools, including kriging interpolation—a statistical method used to predict values at unsampled locations—to map soil contamination and identify hotspots. Each participant received an analysis of trace elements in their soil, along with practical remediation advice. For example, recommendations often included simple and accessible strategies such as adding compost or mulch to reduce exposure to contaminants—materials that could be sourced locally and applied with minimal effort. The researchers shared their findings through user-friendly maps, social media posts, and newsletters to keep the community engaged and informed.

Students also brought samples from their homes, many of which lacked dedicated garden spaces and instead had open yards or shared green areas—conditions often seen in more economically disadvantaged communities with limited access to gardening infrastructure. They also tested local playgrounds and parks. While community interest was generally high, a few schools declined to participate, expressing concern about discovering contamination and uncertainty around how to respond. This hesitation persisted despite reassurances that their lab would provide simple, low-cost remediation strategies, such as building raised beds and using compost and mulch. These responses suggest a pattern of reluctance tied to limited capacity or perceived responsibility to act on the findings.

Overall, participants appreciated the follow-through and found the information interesting and helpful. Many expressed their gratitude through email replies after receiving their results or in person during community events and follow-up conversations. This informal feedback underscored the importance of community involvement in urban soil science and demonstrated the value of returning results in a clear and actionable way.

#### Personal experiences in New York City vs. Lafayette, Louisiana

During outreach efforts, Anna observed notable differences in community responses between New York City and Lafayette, Louisiana. In NYC, reactions typically fell into two categories: some residents were eager to learn and test their soil but found scientific materials overly technical and difficult to access, while others were reluctant to test out of concern that discovering contamination might negatively affect property values. In contrast, many residents in Lafayette were simply unaware of the risks of soil contamination and had not previously considered soil health at all, making outreach and engagement more challenging. These differences reflect broader variations in environmental awareness, housing market concerns, and public health communication influenced by the local sociodemographic context. In New York City, residents tended to be more attuned to environmental issues, likely due to the city’s industrial history, dense population, and frequent media coverage. Many sought consultations proactively.

In Louisiana, however, engaging communities—particularly adults—proved more difficult, as many were parents balancing childcare responsibilities. To respond to this, the team adapted by designing child-friendly outreach activities that encouraged family participation. Events were held at art festivals, local community gatherings, and university-hosted programs, where children could paint with soil, explore minerals under microscopes, and participate in hands-on learning, while the team engaged parents in conversations about soil sampling, contamination, and testing practices.

### Challenges and opportunities

#### Staying connected with communities

Maintaining strong relationships with communities requires a range of strategies to support continued engagement and accessibility. One foundational approach involved creating a public-facing website optimized for search engines, ensuring that community members could easily find information. Involving undergraduate students in Anna’s classroom-based research further enhanced engagement, as students collected and analyzed soil samples throughout the semester and often shared their findings with family members and friends.

To broaden public outreach, Anna actively utilized social media platforms such as Instagram and YouTube to communicate urban soil science in an accessible and engaging way. Project updates were also regularly posted by her students in local Facebook gardening groups, fostering ongoing dialogue with the community.

As part of the Lafayette study, Holly Heafner developed a visual research digest that included maps and key findings, which she emailed to all participants and linked to the related peer-reviewed article.^8^ The study also received broader visibility through features in the local newspaper and on television, helping raise public awareness and deepen community involvement.

#### Where do we go from now?

In her new role at Purdue University’s College of Agriculture (since Fall 2024), Dr. Anna Paltseva is building on her foundation in urban soil research and community engagement. Purdue is poised to become a hub for advancing urban soil science—integrating public participation and leveraging emerging technologies to support more resilient cities. A key resource in this effort is Urban Soil Guide: A Field and Laboratory Manual,^9^ which offers practical tools for scientists, community organizations, and home gardeners to better understand and manage urban soils.

Looking ahead, Anna aims to develop accessible tools for soil data collection and expand collaborations with local communities and policymakers in Indiana. These efforts build on ongoing research conducted with collaborators in cities like Chicago—where the team is surveying park soils to assess their potential for urban agriculture—and St. Louis, where they are examining the distribution of heavy metals across different landscapes and land management types. Complementing this mission is her digital video library on YouTube (https://www.youtube.com/@annapaltseva), which features not only her own content but also video lectures from leading experts around the world. The channel covers topics such as soil science, environmental science, sustainable infrastructure, and practical soil education—making soil science accessible to everyone from professionals to home gardeners.

Although her time at Purdue has just begun, the path forward holds tremendous potential for innovation, collaboration, and impact across both science and society.

## Acknowledgments

This work was shaped by the mentorship of Dr. Zhongqi (Joshua) Cheng at the CUNY Graduate Center and Brooklyn College. Thanks are due to the Brooklyn College Urban Soils Lab team, as well as Anna’s former students from the University of Louisiana at Lafayette and the Delta Urban Soils Lab, for their assistance in fieldwork and soil testing. Dr. Paltseva extends her special gratitude to Holly Heafner for conducting research on soil heavy metals in Lafayette, LA. A.P.’s research was supported by the 10.13039/100006952Louisiana Board of Regents (LEQSF 2022-24-RD-A-25) and the 10.13039/100000199USDA Cooperative Agreement (NR223A750025C008). Gratitude is also extended to NYC organizations and residents, as well as Lafayette, LA, local government partners and residents, for their participation and collaboration. Special thanks to Springer Nature Group for publishing *The Urban Soil Guide: A Field and Laboratory Manual* and to Purdue University College of Agriculture for the new opportunity and continued support.

## Declaration of generative AI and AI-assisted technologies in the writing process

During the preparation of this work, the author used ChatGPT to improve the readability and language of the manuscript. After using this service, the author reviewed and edited the content as needed and took full responsibility for the content of the publication.
